# The Coordination of Aluminum Sulfate with a Water-Soluble Block Copolymer Containing Carboxyl, Amide, Sulfonic and Anhydride Groups Providing Both Accelerating and Hardening Effects in Cement Setting

**DOI:** 10.3390/molecules29194543

**Published:** 2024-09-25

**Authors:** Zhiyuan Song, Sidra Chaudhary, Zainab Bibi, Yong Wu, Qinxiang Jia, Xiaoyong Li, Weiyi Ouyang, Yang Sun

**Affiliations:** 1 Department of Applied Chemistry, School of Chemistry, Xi’an Jiaotong University, No. 28, Xianning West Road, Xi’an 710049, China; 13038012682@163.com (Z.S.); sidra-ch576@stu.xjtu.edu.cn (S.C.); zainabbibi55@stu.xjtu.edu.cn (Z.B.); specwy@mail.xjtu.edu.cn (Y.W.); qinxiangjia1984@mail.xjtu.edu.cn (Q.J.); lixy6658@xjtu.edu.cn (X.L.); weiyi.ouyang@xjtu.edu.cn (W.O.); 2 Shanxi Jiawei New Material Co., Ltd., Taijia Village, Jiedian Town, Wanrong County, Yuncheng 044200, China; 3 Xi’an Biomass Green Catalysis and Advanced Valorization International Science and Technology Cooperation Base, No. 28, Xianning West Road, Xi’an 710049, China

**Keywords:** aluminum sulfate, block copolymer, accelerator, mortar, cement hydration, setting time

## Abstract

Two water-soluble block copolymers composed of acrylic acid (AA), 2-acrylamido-2-methylpropane sulfonic acid (AMPS), and optionally maleic anhydride (MAH) were synthesized through ammonium persulfate-catalyzed free radical polymerization in water. The introduction of aluminum sulfate (AS) into the resulting mixtures significantly reduced the setting times of the paste and enhanced the mechanical strength of the mortar compared to both the additive-free control and experiments facilitated solely by pure AS. This improvement was primarily attributed to the inhibition of rapid Al^3+^ hydrolysis, which was achieved through coordination of the synthesized block copolymers, along with the formation of newly identified hydrolytic intermediates. Notably, the ternary copolymer (AA–AMPS–MAH) exhibited superior performance compared to that of the binary copolymer (AA–AMPS). In the early stages of cement setting, clusters of ettringite (AFt) were found to be immobilized over newly detected linkage phases, including unusual calcium silicate hydrate and epistilbite. In contrast to the well-documented role of polymers in retarding cement hydration, this study presents a novel approach by providing both accelerating and hardening agents for cement setting, which has significant implications for the future design of cement additives.

## 1. Introduction

To this day, cement remains one of the most widely used construction materials. Initially, it was hydrated into an inorganic hydrogel known as hydrated tricalcium silicate (C-S-H, 3CaO·SiO_2_·3H_2_O), which then bound together various concrete components, such as bricks, wood, and sand, tightly and permanently, ensuring the durability of the structures for many years [1,2].

The rapid setting and hardening of OPC (Ordinary Portland Cement, also known as Standard Cement in China)-based concrete was crucial in cold regions and for constructing tunnels, bridges, and roadways. Without it, these structures could fail quickly due to prolonged setting times and slow development of mechanical strength. To address this issue, sprayed concrete (shotcrete) was developed [3]. The primary difference between conventional and sprayed concrete lies in the use of accelerators. However, accelerators can sometimes lead to long-term strength loss (after 28 days) and result in issues, such as shrinkage and durability [3].

Over time, three main series of accelerators have been developed for both market and engineering applications. The first series includes setting and hardening accelerators. Setting accelerators focus on reducing the hydration time of concrete while hardening accelerators are designed to enhance early strength [4]. The second series, addressing concerns about the corrosion of steel in concrete due to chloride ions, includes both chloride-containing and non-chloride accelerators. Chloride-based accelerators, such as CaCl_2_, NaCl, and LiCl, are effective in promoting early-stage hydration [5]. However, due to issues such as excessive dosage, high cost, and the corrosive effects on steel reinforcement [5], their use has become less common. Consequently, non-chloride accelerators have gained prominence. For example, the combination of Ca(NO_3_)_2_ and NaNO_3_ has been effective in accelerating the cementing process while simultaneously improving early strength [6].

Lastly, it was discovered that excessive amounts of alkaline metal ions could trigger alkali-aggregate reactions during cement setting, leading to expansion and a significant loss of strength [4]. As a result, alkali-free accelerators containing 1% or less alkaline content have become more popular in both market and engineering applications compared to their alkali-containing counterparts [4].

In recent years, aluminum sulfate (AS, Al_2_(SO_4_)_3_·18H_2_O) has emerged as a prominent and widely used material in the field of alkali-free accelerators. During the hydration of Ordinary Portland Cement (OPC), the pH gradually increases, causing Al^3+^ ions from AS to hydrolyze into [Al(OH)_4_]^−^. This species then reacts with Ca^2+^ and SO_4_^2−^ ions, leading to the formation of C_3_A (tricalcium aluminate, 3CaO·Al_2_O_3_) [7]. Further hydration of C_3_A with gypsum (CaSO_4_·2H_2_O) results in the formation of AFt (ettringite, calcium sulfoaluminate hydrate, 3CaO·Al_2_O_3_·3CaSO_4_·32H_2_O), which plays a crucial role as an adhesive in enhancing mechanical strength [7]. In OPC-based concretes, C-S-H (calcium silicate hydrate) bound SiO_2_, lime, and other components, typically form a two-dimensional layered structure that is not as robust as desired. The formation of AFt significantly improves this linkage by strengthening the material in an additional dimension [8].

However, while AS facilitates the hydration of C_3_A and the subsequent formation of AFt, the rapid and substantial accumulation of AFt on the surface of C_3_S can inhibit its hydration of C_3_S due to inadequate water dispersion [8]. During this process, the morphology of AFt transitions from rounded masses to prismatic needles [9]. Consequently, to prevent the agglomeration of AFt on the C_3_S surface, it is essential to regulate the rapid hydrolysis of pure AS into Al^3+^ and [Al(OH)_4_]^−^ by incorporating functional additives [10,11,12,13].

The design and application of polymers in cement hydration have garnered ongoing interest due to their structural versatility and relatively low cost [14]. In practice, the most commonly used polymers function as retarders or water reducers. Their dispersion in water and subsequent attachment to the surfaces of cement particles inhibit rapid hydration while maintaining high fluidity in mortar, thereby enhancing castability [15]. For instance, the incorporation of rubber latexes as additives in paving cement has improved various properties, including workability, adhesion to substrates, anti-bleeding characteristics, flexural and tensile strength, ductility, cracking resistance, impermeability, and reduced shrinkage [16]. Currently, EVA (ethylene-vinyl acetate copolymer) stands out as the most widely utilized polymer in concrete engineering, significantly enhancing the tensile bond strength, flexural strength, and toughness of hardened concrete [17].

There are numerous examples of using polymers as retarders or superplasticizers in the cementing process. For instance, an amphoteric retarder derived from the copolymerization of AMPS (2-acrylamido-2-methylpropane sulfonic acid), IA (itaconic acid), DMC (2-(methacryloyloxy)ethyltrimethylammonium chloride), and TPEG (methylallyl polyethenoxy ether), has demonstrated an excellent retarding effect at high temperatures for long-standing cementing applications [18]. This makes it suitable for deep-well construction [18]. Additionally, PCE (polycarboxylate ether) superplasticizers have been found to enhance the initial dissolution rate of cement and subsequently inhibit hydration through complexation with Ca^2+^, thereby exhibiting significant plasticizing properties [19].

There are notable examples of organic polymers being utilized as accelerators in cementing. For instance, the combination of lignosulfonate with CaCl_2_ has demonstrated significant accelerating effects on cement hydration, resulting in high compressive strength after 28 days. However, this accelerator did not effectively prevent the deterioration of the blended cement in corrosive environments [20]. Additionally, polyacrylamide, a linear water-soluble polymer, plays a crucial role when added to cement slurry. The molecules of polyacrylamide linked together and adsorbed onto two or more cement particles, forming bridges that facilitate the formation of a cohesive structure. This interaction increases resistance to particle movement, thereby enhancing the viscosity of the slurry and accelerating the cementing setting [21,22].

However, there is a strong demand for cementing solutions that offer both short setting times and high mechanical strength, especially in engineering applications, such as the use of sprayed concrete (shotcrete) under high pressure and velocity for tunnel construction. In this context, block copolymers composed of various monomers with different functional groups are of particular interest. Each functional group imparts unique properties, and their combination can lead to unexpected and highly effective improvements in the cementing performance.

Initially, the primary role of the AA (acrylic acid) monomer was attributed to its carboxyl group, which prevented the agglomeration of Ca^2+^ during cement hydration, thus demonstrating excellent salt tolerance [15]. Additionally, the carboxyl group creates an acidic environment that inhibits rapid and excessive hydrolysis of Al^3+^, thereby naturally accelerating the cementing process [23].

Furthermore, AMPS (2-acrylamido-2-methylpropane sulfonic acid) contains both sulfonic acid and amide groups. The sulfonic acid group provides high water solubility and good thermal stability, while the amide group helps control fluid loss and can be hydrolyzed at high temperatures, thereby enhancing the rheological performance [24].

Moreover, the MAH (maleic anhydride) monomer contributes to water reduction, which improves paste fluidity and accelerates the setting time [25]. Given the diverse properties of these functional monomers, it is intriguing to explore whether copolymerization of these groups could achieve both rapid setting and effective hardening in cementing applications.

To enhance the performance of AS-induced cement hydration, two block copolymers such as AA–AMPS and AA–AMPS–MAH were synthesized via (NH_4_)_2_S_2_O_8_-initiated free radical polymerization in an aqueous solution. These copolymers were then combined with AS to serve as accelerators in the hydration of Ordinary Portland Cement (OPC). This study involved measuring both the setting times of the paste and the mechanical properties of the mortar as well as exploring parameters such as the type and dosage of the accelerator. Additionally, characterization was conducted to understand the underlying mechanisms. This research aimed to contribute to the development of new accelerators with hardening effects, particularly in the context of sprayed concrete applications.

## 2. Results and Discussion

### 2.1. Characterization of Block Copolymers and Accelerators

**Figure 1:**
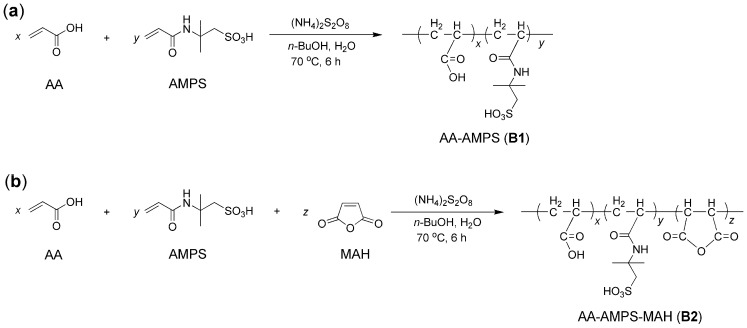
Synthesis of block copolymers: (**a**) AA–AMPS (**B1**) and (**b**) AA–AMPS–MAH (**B2**).

Synthesis of block copolymers were shown in Figure 1. The *X* and *α* values of the prepared block copolymers are shown in Table 1. (NH_4_)_2_S_2_O_8_ seemed to be an effective initiator for the aqueous free radical polymerization of AA, AMPS, and MAH at moderate temperatures (Figure 1) [26], and *α* stemmed from the polymerization of AA, AMPS, and MAH was slightly better than that of AA and AMPS (Table 1). It was previously reported that MAH showed high activity in copolymerization with olefinic compounds, leading to uniform polymeric microspheres [27], which may promote the copolymerization of AA and AMPS in this work (Figure 1).

**Figure 2:**
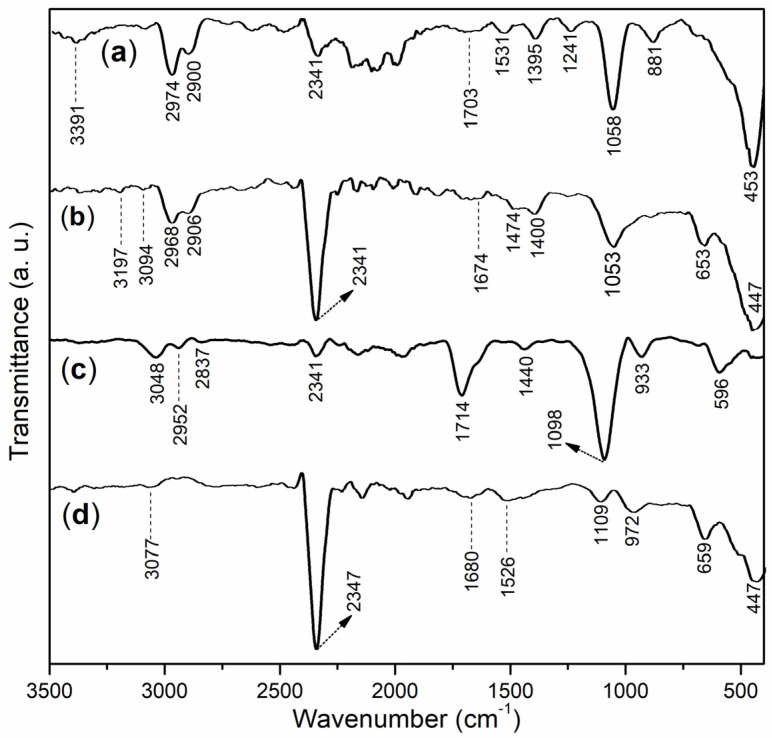
FT-IR spectra of block copolymers and accelerators: (**a**) **B1**; (**b**) **B1a**; (**c**) **B2**; (**d**) **B2a**.

The FT-IR spectra of all the block copolymers (**B1** and **B2**, Section 3.3) and their corresponding accelerators (**B1a** and **B2a**, accordingly, Section 3.5) are presented in Figure 2. In Figure 2a, a moderate broadband at 3391 cm^−1^ for **B1** corresponds to the O–H stretching vibrations of carboxyl groups from acrylic acid (AA) and sulfonic acid groups from AMPS [28]. It has been previously reported that hydrogen-bonded hydroxyl groups exhibit lower intensities and broader FT-IR peaks compared to non-hydrogen-bonded groups [29]. The peaks at 2974 and 2900 cm^−1^ were indicative of the anti-symmetric and symmetric stretching of the C–H bonds in the methyl groups of AMPS in **B1**, respectively [28]. Additionally, the peak at 2341 cm^−1^ was associated with the vibration of CO_2_ (due to air interference).

Furthermore, **B1** showed a small band at 1703 cm^−1^, probably indicating C=O stretching of the carboxyl group on AA [30]. Meanwhile, the small band that occurred at 1531 cm^−1^ was characteristic of the C=O vibration of the amide group on AMPS [30]. Moreover, there was one small peak at 1395 cm^−1^, which could be ascribed to the C–H bending vibration of the **B1** framework (Figure 1). The following two peaks appeared at 1241 and 1058 cm^−1^ and were attributed to the anti-symmetric and symmetric stretching vibrations of the sulfonic acid group on AMPS [30]. Additionally, **B1** also showed peaks at 881 and 453 cm^−1^, which could be attributed to the residual effects of the sulfonic acid group on AMPS.

The small band observed at 3391 cm^−1^ in **B1** notably diminished and shifted to 3197 cm^−1^ after **B1** was mixed with AS, resulting in the formation of accelerator **B1a** (Figure 2b vs. Figure 2a). This shift suggested that the O–H units of the carboxyl and sulfonic acid groups in **B1** coordinated with Al^3+^ [31]. Additionally, when comparing **B1** with **B1a**, a weak peak at 1674 cm^−1^ was detected in **B1a**, which was significantly lower than the 1703 cm^−1^ peak observed in **B1** (Figure 2b vs. Figure 2a). This change further indicated that the C=O unit of the carboxyl group in **B1**’s AA component also coordinated with Al^3+^ [32]. The same phenomenon was observed for the C=O unit of the amide on the AMPS of **B1** (1474 vs. 1531 cm^−1^, Figure 2b vs. Figure 2a).

Moreover, the peak observed at 1058 cm^−1^ on **B1** red-shifted to 1053 cm ^−1^ (Figure 2b vs. Figure 2a), suggesting that the S=O unit of the amide group on AMPS was attached to Al^3+^. In addition, there was a new peak at 447 cm^−1^ on **B1a**, which was different from that at 453 cm^−1^ on **B1** (Figure 2b vs. Figure 2a), which could be attributed to the Al–O stretching vibration of [Al(OH)_4_]^−^ originating from the hydrolysis of Al^3+^ during the preparation of the accelerator (Section 2.5).

The **B2** showed a moderate peak at 3028 cm^−1^ (Figure 2c), which summarized the effects of the O–H groups of both carboxyl groups on AA and sulfonic acid groups on AMPS. There were two following peaks occurred at 2952 and 2837 cm^−1^ (Figure 2c), mainly owing to anti-symmetric and symmetric stretching of the C–H bond of the methylene group on **B2**, which were different from those found at 2974 and 2900 cm^−1^ on **B1**, obviously due to the copolymerization of MAH (Figure 1b). The next peak appeared at 1714 cm^−1^, compared to 1703 cm^−1^ on **B1** (Figure 2c vs. Figure 2a), reflecting the influence of C=O originating from the carboxyl group on AA, as well as that of the cyclic lactone on MAH on **B2**. Furthermore, **B2** showed another weak peak at 1400 cm^−1^, indicating C–H bending of the **B2** framework (Figure 1b). In addition, the larger peak formed at 1098 cm^−1^ clearly indicates the symmetric stretching of the sulfonic acid group on the AMPS of **B2**.

On the other hand, the comparison of **B2a** with **B2** showed a very similar tendency to that of **B1a** with **B1**, similar to 3077 (weak and broad) vs. 3048, 1680 vs. 1714, and 972 vs. 1098 (Figure 2d vs. Figure 2c), clearly indicating that the O–H groups of carboxyl groups on AA and sulfonic acid groups on AMPS, C=O originating from carboxyl group on AA, C=O of cyclic lactone MAH, and S=O unit of amide group on AMPS were all attached to Al^3+^.

### 2.2. Effects of Accelerators

The IST and FST of the cement paste, along with the compressive and flexural strengths of the cement mortar, are summarized in Table 2. Above all, in the absence of any accelerator, the IST and FST of the cement paste were prolonged, and the compressive and flexural strengths of the mortar developed very slowly during 6 h, 24 h, and 28 d (entry 1, Table 2). When pure AS was introduced as an accelerator, both the IST and FST were shortened significantly, and the compressive and flexural strengths improved at 6 h, 24 h, and 28 d (entries 2 vs. 1, Table 2). However, the present IST and FST (18.75 and 36.74 min, entry 2, Table 2) could not match the requirements of Chinese standard GB/T 35159-2017 (IST ≤ 5 min, FST ≤ 12 min). The use of **B1** as an accelerator could slightly decrease the setting times and concurrently improve the mechanical strength compared to the blank experiment (entries 3 vs. 1, Table 2), probably owing to the acidic nature of **B1** in the cement setting, similar to the effect of inorganic acids as accelerators [4]. 

The application of **B1a** sharply decreased both the IST and FST of paste in comparison with those of AS and **B1**, and promoted the development of both compressive and flexural strengths (entries 4 vs. 2 and 3, Table 2). Actually, the same tendency was found in the utilization of **B2a** (entries 7 vs. 2 and 3, Table 2). Therefore, this series of accelerators promoted the setting and hardening of cement together.

Herein, coordination of the block copolymer to Al^3+^ may inhibit the agglomeration and hydrolysis of Al^3+^ into unreactive Al(OH)_3_ or Al_2_O_3_, subsequently stabilizing the formation of [Al(OH)_4_]^−^, which accelerates the occurrence of C_3_A (precursor of AFt), leading to faster setting [4,7]. At the same time, the block copolymers may act as structure-directing agents and contribute to the ordered arrangement of AFt on the surface of C_3_S [11].

### 2.3. Effects of Maleic Anhydride Monomer and Accelerator Dosage

With the accelerator, the dosage was fixed at 7 wt.%, **B2a** showed similar IST as well as decreased FST than **B1a**, and both compressive and flexural strengths of mortar derived from **B2a** were much higher than those from **B1a** (entries 8 vs. 5, Table 2). Furthermore, **B2a** showed higher *R*_28_ and *R*_r, 90_ values than those of **B1a** (Table 3). These results indicated that the incorporation of MAH into the AA–AMPSs chain may play a key role in the coordination between Al^3+^ and the block copolymer, which affected the formation and arrangement of C_3_A and the subsequent AFt. It was recently reported that MAH can be copolymerized with other olefinic compounds into uniform polymeric microspheres through self-stabilized precipitation polymerization [27]. Obviously, this unique property of MAH may contribute to the fast setting and strength development in cementing.

The accelerator dosage also facilitated cement setting. When **B1a** was used as an accelerator, a dosage of 7 wt.% performed much better than 6 wt.% and 8 wt.% (entries 5 vs. 4 and 6, Table 2), and the same tendency was found for the cementing accelerated by **B2a** (entries 8 vs. 7 and 9, Table 2). This result may be related to the presence of organic residues in the cement paste and mortar.

### 2.4. Analytical Insights into Cementing Process Facilitated by Accelerator

#### 2.4.1. Composition of Cement and Accelerators

**Figure 3:**
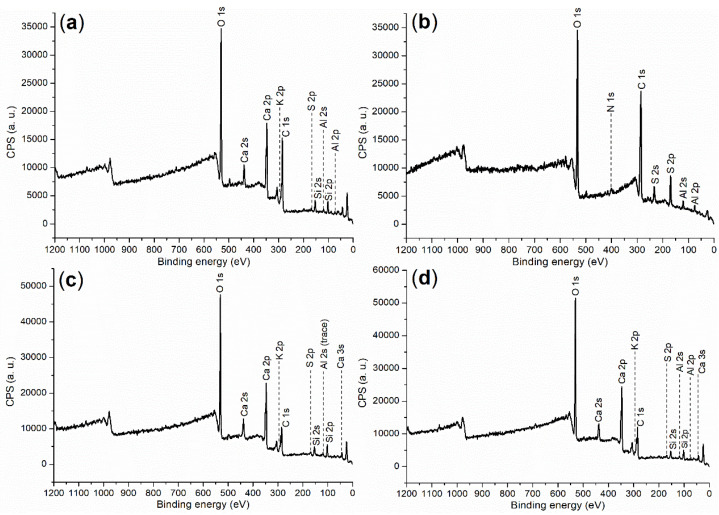
XPS survey scan: (**a**) cement; (**b**) **B2a**; (**c**) cement mortar of 24 h, entry 5, Table 2; (**d**) cement mortar of 24 h, entry 8, Table 2.

In order to further understand the cementing process facilitated by the accelerator, comprehensive characterizations were carried out to provide clear illustrations. First of all, Table 4 shows the composition and ignition loss of cement, corresponding to those of typical OPC [33]. For cement (raw material) and hydration products, XPS survey scans are listed in Figure 3, while binding energy and atomic composition are in Table 5. Most elements of cement detected by ICP–OES can be found on the cement surface through XPS (Figure 3a vs. Table 4), but the XPS signals of Fe, Na, and Mg were too weak to be collected (Figure 3a; entry cement, Table 5), mainly due to their low contents.

**Figure 4:**
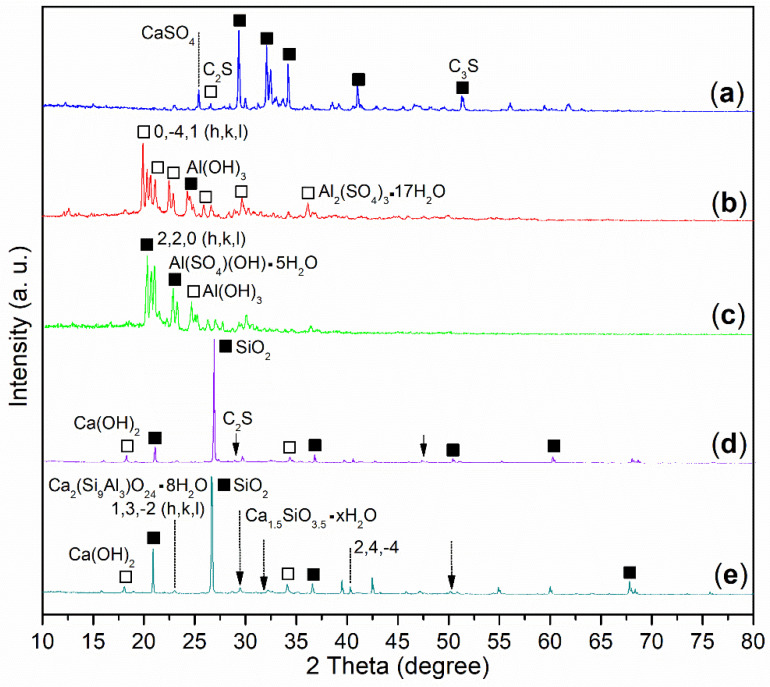
Wide-angle XRD spectra: (**a**) cement; (**b**) **B1a**; (**c**) **B2a**; (**d**) mortar of 24 h, entry 5, Table 2; (**e**) mortar of 24 h, entry 8, Table 2.

On the other hand, there were three phases in the wide-angle XRD of the cement, including C_3_S (dark cubes, Figure 4a; 3CaO·SiO_2_, PDF No. 31-0301), C_2_S (white cube, Figure 4a; 2CaO·SiO_2_, PDF No. 31-0302), and CaSO_4_ (dotted line, Figure 4a; PDF No. 37-0184).

**Figure 5:**
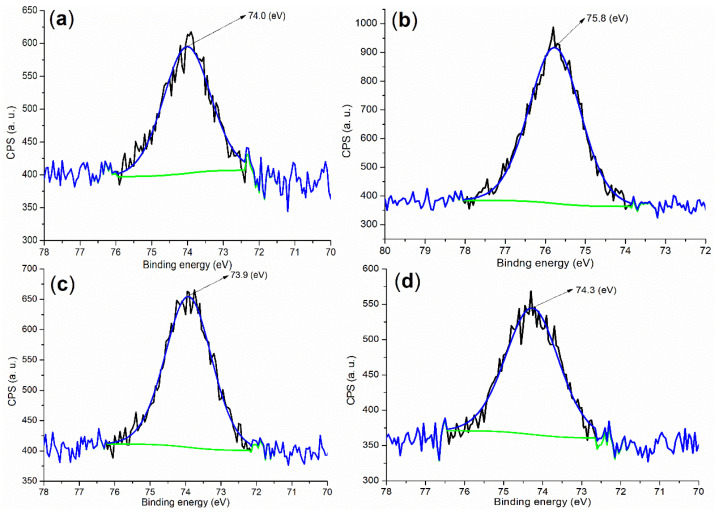
XPS measurements of the Al 2p region: (**a**) cement; (**b**) **B2a**; (**c**) mortar of 24 h, entry 5, Table 2; (**d**) mortar of 24 h, entry 8, Table 2.

Next, it seemed necessary to determine the chemical state of Al during cementing. The binding energy of the Al 2p photoelectron coming from fresh cement was centered at 74.0 eV as shown in Figure 5a, which is much higher than that of metallic Al (72.6 Â eV) [34] but lower than the Al^3+^ of the Al_2_O_3_ phase (75.9 Â eV) [35], probably characterizing the tetrahedral Al^3+^ of the Al(OH)_3_ phase in the cement.

On the other hand, **B2a** showed the XPS peak of Al 2p at 75.8 eV (Figure 5b), a little lower than that of Al^3+^ of Al_2_O_3_ (75.9 eV) [35], meaning some new Al^3+^-containing species occurred. In association with XRD, there were two phases, Al(SO_4_)(OH)·5H_2_O (black cubes, Figure 4c; rostite, PDF No. 41-1382; the peak at 100% intensity at 2*θ* = 20.860 ° with h, k, l of 2, 2, 0), and Al(OH)_3_ (white cube, Figure 4c; PDF No. 26-0025) on **B2a**. The first phase indicated that AS was hydrolyzed stepwise, while the second phase indicated that the remaining AS was completely hydrolyzed.

In comparison, **B1a** exhibited diffraction systems of Al_2_(SO_4_)_3_·17H_2_O (white cubes, Figure 4b; alunogen, PDF No. 26-1010; the peak of 100% intensity at 2*θ* = 19.906 ° with h, k, l of 0, −4, 1) and Al(OH)_3_ (dark cube, Figure 4b; PDF No. 37-1377). The former phase illustrated that AS was almost untouched, while the latter was characteristic of hydrolyzed Al^3+^.

Evidently, the roles of **B1** and **B2** appear to be different in the formation of the accelerator. Therefore, the incorporation of MAH as a monomer increased accelerator activity to a large extent (entries 8 vs. 5, Table 2). Additionally, Figure 5c,d showed two peaks at 73.9 and 74.3 eV, respectively, lower than 75.8 eV on **B2a**, indicating Al^3+^ may come into the Al_2_O_3_ phase rather than remain in the accelerator as cementing continued.

#### 2.4.2. Composition of Hydrated Mortars

**Figure 6:**
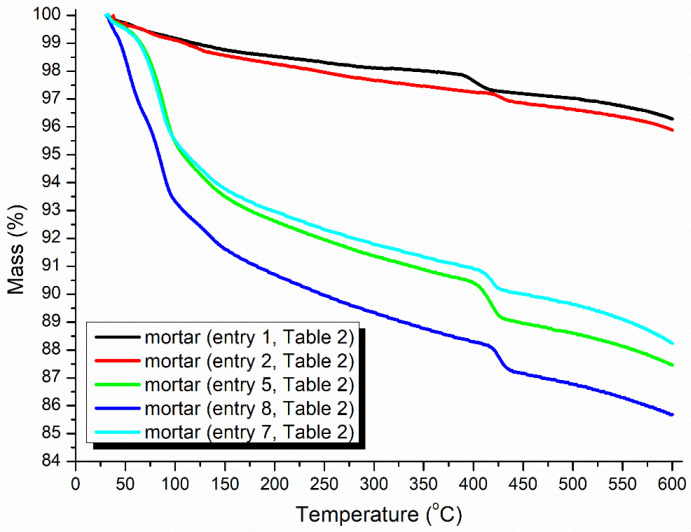
Thermogravimetric analysis (TGA) of the mortars (24 h, Table 2).

The TGA of the mortar may provide clues for illustrating the accelerating and hardening effects of the accelerators (Figure 6). At first, the red line was always located below the black line at 30–600 °C, indicating that the loading of pure AS as an accelerator resulted in more crystalline water and volatile species than in the blank experiment, but pure AS provided faster setting times and higher mechanical strengths (entries 2 vs. 1, Table 2).

Next, the use of **B1a** as an accelerator induced much more crystalline water and organic species in the mortar than AS (green vs. red, Figure 6), corresponding to a decreased setting time and enhanced strength (entries 5 vs. 2, Table 2). The blue line was continuously located below the green line at 30–600 °C (Figure 6), indicating that **B2a** introduced more crystalline water and organic species than **B1a** at the same dosage (7 wt.%).

The larger the dosage of the accelerator loaded, the more volatiles contained in the mortar (blue vs. slightly blue, Figure 6), leading to a better setting behavior (entries 8 vs. 7, Table 2). However, excessive loading of **B2a** depressed the setting conversely (entries 9 vs. 8, Table 2), indicating that the accelerator dosage was highly sensitive to the setting behavior.

**Figure 7:**
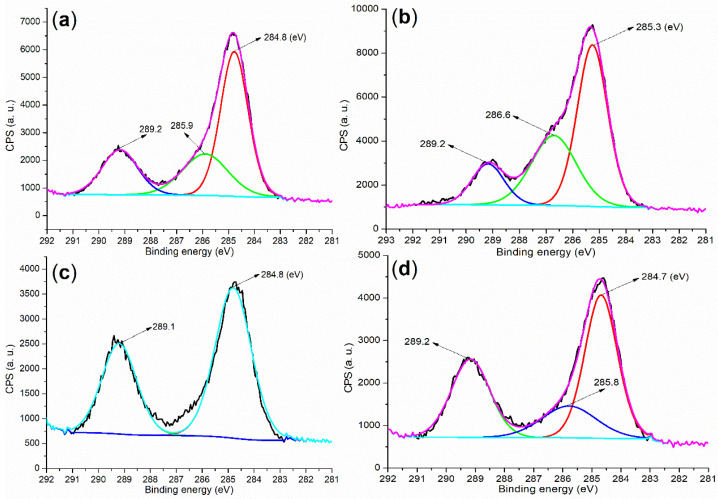
XPS measurements of the C 1s region: (**a**) cement; (**b**) **B2a**; (**c**) mortar of 24 h, entry 5, Table 2; (**d**) mortar of 24 h, entry 8, Table 2.

Based on the data obtained thus far, there has been a growing interest in detecting organic residues in hydrated mortar. Initially, three distinct peaks were observed at 284.8, 285.9, and 289.2 eV in the cement, corresponding to carbons of the C (sp^3^ configuration, saturated)–H group, C–O bond, and carboxyl group, respectively (Figure 7a) [36]. These peaks can be attributed to the presence of small organic molecules residual in the cement. In contrast, **B2a** exhibited three C 1s peaks at 285.3, 286.6, and 289.2 eV (Figure 7b), which are associated with carbons from the C–O bond, C–N bond in amides, and carboxyl group [36]. These features primarily originated from the functional groups in the **B2** backbone (Figure 1).

Furthermore, the mortar induced by **B2a** exhibited three peaks at 284.7, 285.8, and 289.2 eV (Figure 7d), which were significantly closer to those observed in cement than to those in **B2a**. Additionally, after hydration facilitated by **B1a**, the resulting mortar displayed two peaks at 284.8 and 289.1 eV (Figure 7c), which are also in close proximity to those found in cement, indicating the presence of saturated C–H and carboxyl groups. These observations suggested that most of the water-soluble block copolymer may have been washed away during hydration, while the remaining residues appeared to consist of small organic molecules.

Given that **B2a**, at the same dosage of 7 wt.% introduced more crystalline water and organic species compared to **B1a** (blue vs. green, Figure 6) and exhibited significantly shorter setting times and higher mechanical strengths in cementing (entries 8 vs. 5, Table 2), it appeared that **B2** (the organic backbone of **B2a**) may function as an active template in mortar hydration.

In association with XRD, there were three components in the **B1a**-facilitated mortar (24 h), including SiO_2_ (dark tubes, Figure 4d; quartz low, PDF No. 65-0466), Ca(OH)_2_ (white tubes, Figure 4d; Portlandite, PDF No. 04-0733), and unreacted C_2_S (arrow, Figure 4d; 2CaO·SiO_2_, PDF No. 49-1672). It seemed that the weight loss of this mortar (TGA) originated from the dehydration of Ca(OH)_2_, as well as the release of organic **B1** residues (green line, Figure 6).

However, the **B2a**-induced mortar (24 h) showed four phases: SiO_2_ (dark tubes, Figure 4e; quartz, PDF No. 46-1045), Ca(OH)_2_ (white cubes, Figure 4e; Portlandite, PDF No. 04-0733), Ca_1.5_SiO_3.5_·xH_2_O (dotted arrows, Figure 4e; calcium silicate hydrate, PDF No. 33-0306), and Ca_2_(Si_9_Al_3_)O_24_·8H_2_O (dotted lines, Figure 4e; epistilbite, PDF No. 39-1381) as well. The weight loss of the **B2a**-induced mortar (24 h) at 30–600 °C can be attributed to the dehydration of Ca(OH)_2_, Ca_1.5_SiO_3.5_·xH_2_O, and Ca_2_(Si_9_Al_3_)O_24_·8H_2_O (30–200 °C) along with the release of organic **B2** residues (200–600 °C) [37].

**Figure 8:**
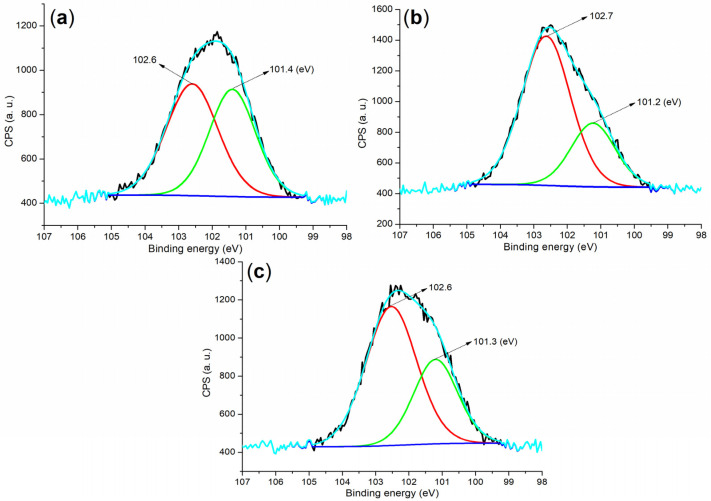
XPS measurements of the Si 2p region: (**a**) cement; (**b**) mortar for 24 h, entry 5, Table 2; and (**c**) mortar for 24 h, entry 8, Table 2.

The Si 2p regions of the cement and mortar provided valuable insights into the formation of new phases during hydration. Initially, cement exhibited two peaks at 102.6 eV and 101.4 eV (Figure 8a), which corresponded to silicon in SiO_2_ and C_3_S, respectively (Figure 4a) [38]. After 24 h of hydration under **B1a**, the Si 2p peak associated with Si in C_3_S significantly diminished and shifted by 0.2 eV compared to the peak observed in the cement (Figure 8b vs. Figure 8a). This shift indicated that a substantial portion of C_3_S was transformed into hydrated products, while C_2_S remained (Figure 4d).

Furthermore, the Si 2p peaks observed in the **B2a**-facilitated mortar after 24 h, at 102.6 and 101.3 eV (Figure 8c), were similar to those found in the **B1a**-facilitated mortar in terms of binding energy and peak intensity. These peaks correspond to silicon in SiO_2_ and other silicon-containing phases, such as Ca_1.5_SiO_3.5_·xH_2_O and Ca_2_(Si_9_Al_3_)O_24_·8H_2_O (Figure 4e).

**Figure 9:**
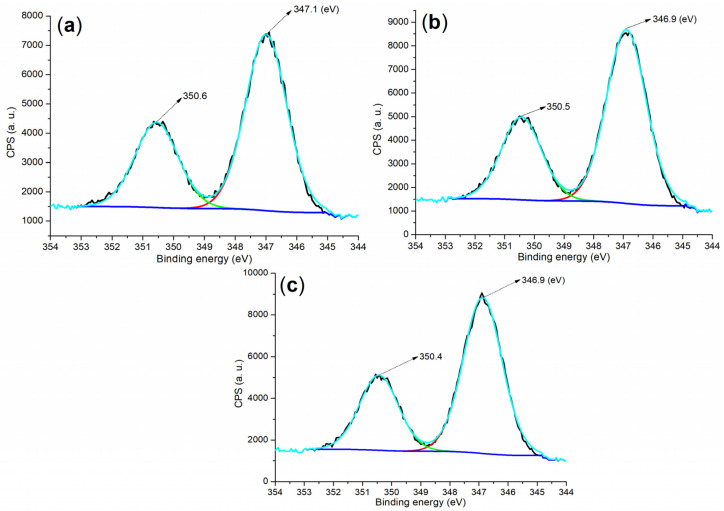
XPS measurements of the Ca 2p region: (**a**) cement; (**b**) mortar for 24 h, entry 5, Table 2; (**c**) mortar for 24 h, entry 8, Table 2.

To further analyze the composition of the hydrated mortar, the Ca 2p regions of both the raw material and the hydrated mortar were examined. As depicted in Figure 9a, the cement displayed two peaks at 350.6 and 347.1 eV, corresponding to Ca 2p_1/2_ and 2p_3/2_ photoelectrons, respectively [39]. These peaks are characteristic of Ca^2+^ in C_3_S, C_2_S, and CaSO_4_ (Figure 4a). In contrast, both **B1a**- and **B2a**-facilitated mortars exhibited lower binding energies for the Ca 2p_1/2_ and 2p_3/2_ photoelectrons compared to the cement (Figure 9b,c vs. Figure 9a), indicating changes in the calcium-containing components (Figure 4d,e vs. Figure 4a).

**Figure 10:**
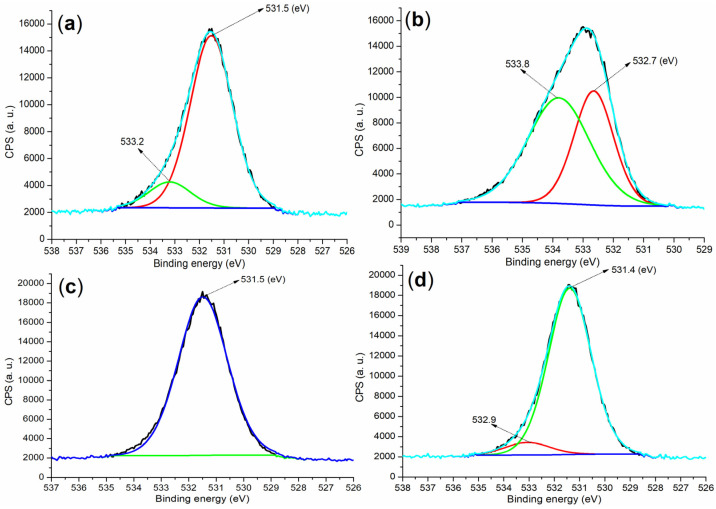
XPS measurements of the O 1s region: (**a**) cement; (**b**) **B2a**; (**c**) mortar of 24 h, entry 5, Table 2; (**d**) mortar of 24 h, entry 8, Table 2.

From the perspective of anions, XPS analysis of the O 1s regions in the raw materials and hydrated mortars can further corroborate the findings of cation detection. Specifically, the cement sample displayed two peaks at 533.2 eV and 531.5 eV (Figure 10a), which were attributed to the oxygens in CaSO_4_ and C_2_S (the former) and C_3_S (the latter) (Figure 4a) [40]. In contrast, the **B2a** showed peaks at 533.8 eV and 532.7 eV (Figure 10b), indicative of oxygens in OH^−^ and H_2_O (the former) and SO_4_^2−^ (the latter) (Figure 4c). Post-hydration, the mortar prepared using **B1a** exhibited an O 1s peak at 531.5 eV (Figure 10c), predominantly corresponding to oxygen in the SiO_2_ phase (Figure 4d). However, the mortar produced with **B2a** presented an additional peak at 532.9 eV compared to the **B1a** sample (Figure 10d vs. Figure 10c), which aligned with the formation of new phases such as Ca_1.5_SiO_3.5_·xH_2_O and Ca_2_(Si_9_Al_3_)O_24_·8H_2_O (Figure 4e).

#### 2.4.3. Morphology of Hydrated Mortars

**Figure 11:**
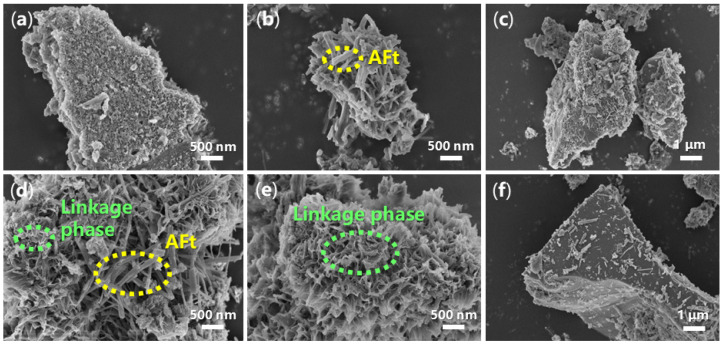
SEM images of the mortar: (**a**) mortar of entry 1, Table 2 (24 h, magnification 20,000×); (**b**) mortar of entry 5, Table 2 (24 h, 20,000×); (**c**) mortar of entry 5, Table 2 (28 d, 10,000×); (**d**) mortar of entry 8, Table 2 (24 h, 20,000×); (**e**) mortar of entry 8, Table 2 (24 h, 20,000×); (**f**) mortar of entry 8, Table 2 (28 d, 10,000×).

From another point of view, the morphology of hydrated mortar may provide more information on the cementing process. In Figure 11a, the accelerator-free mortar shows a layered and porous appearance with a size of several square micrometers. When **B1a** was loaded as an accelerator, there were many clusters composed of fibers with lengths of 500–800 nm (Figure 11b), probably originating from AFt [9]. However, the fiber morphology of the mortar derived from **B2a** appeared much longer (about 500–2000 nm) and slimmer than that from **B1a** (Figure 11d vs. Figure 11b), owing to the different structures of **B1** and **B2** (Figure 1).

Meanwhile, flower-like mesoporous structures were observed on the external surface of the fiber clusters of the mortar stemming from **B2a** (Figure 11e), which may be attributed to the formation of Ca_1.5_SiO_3.5_·xH_2_O and Ca_2_(Si_9_Al_3_)O_24_·8H_2_O (Figure 4e). These newly-formed phases may not only act as sturdy linkers, leading to better setting time and mechanical strength of the mortar (24 h, entries 8 vs. 5, Table 2), but also give a much denser mortar material after incubation for 28 days (Figure 11f vs. Figure 11c).

### 2.5. Proposed Mechanism for Cement Hydration Facilitated by Accelerator **B2a**

**Figure 12:**
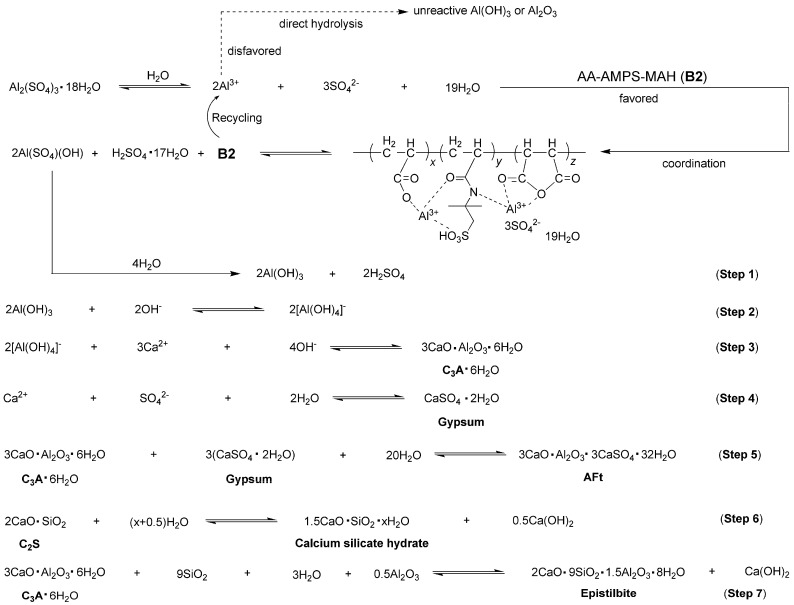
Proposed mechanism for cement hydration facilitated by accelerator **B2a**.

Based on the results obtained so far, the mechanism of cement hydration facilitated by **B2a** can be summarized in Figure 12. Initially, the AS was ionized in water and promptly coordinated by **B2**. This coordination was preferred over the direct hydrolysis of Al^3+^ into less reactive Al(OH)_3_ or Al_2_O_3_ due to the low solubility of AS (36.5 g AS vs. 100 g H_2_O at 20 °C) [41].

Furthermore, the coordination intermediate decomposed into Al(SO_4_)(OH), which was then hydrolyzed to form active Al(OH)_3_ (Step 1, Figure 12). Al(OH)_3_ was formed through the successive substitution of SO_4_^2−^ with OH^−^ from Al(SO_4_)(OH), thereby preventing the agglomeration and precipitation of Al^3+^. Subsequently, the resulting Al(OH)_3_ reacted with OH^−^ to produce [Al(OH)_4_]^−^, which then reacted with Ca^2+^ to form C_3_A (Steps 2–3, Figure 12). Upon the formation of gypsum, C_3_A and gypsum underwent hydrolysis together, ultimately leading to the formation of AFt (Steps 4–5, Figure 12).

Moreover, the C_2_S phase of cement (2CaO·SiO_2_, white cube, Figure 4a) hydrated into calcium silicate hydrate (Ca_1.5_SiO_3.5_·xH_2_O, dotted arrows, Figure 4e) and Ca(OH)_2_ (white cubes, Figure 4e), as illustrated in Step 6 of Figure 12. Concurrently, C_3_A·6H_2_O from Step 4 further reacted with SiO_2_, H_2_O, and Al_2_O_3_ to form epistilbite (Ca_2_(Si_9_Al_3_)O_24_·8H_2_O, dotted lines, Figure 4e) along with additional Ca(OH)_2_ (white cubes, Figure 4e), as depicted in Step 7 of Figure 12.

## 3. Experimental Section

### 3.1. Starting Materials

The AS (aluminum sulfate, Al_2_(SO_4_)_3_·18H_2_O, 99%) was purchased from Shanghai Aladdin Biochemical Technology Co., Ltd. (Shanghai, China). The AA (acrylic acid, 99%), AMPS (2-acrylamido-2-methylpropane sulfonic acid, 98%), MAH (maleic anhydride, 99%), (NH_4_)_2_S_2_O_8_ (ammonium persulfate, 98%), and *n*-BuOH (*n*-butanol, 99%) were purchased from Shanghai Macklin Biochemical Technology Co., Ltd. (Shanghai, China). H_2_SO_4_ (concentrated sulfuric acid, 98%), KBrO_3_ (potassium bromate, 99.5%), KBr (potassium bromide, 99%), HgSO_4_ (mercuric sulfate, 99%), NaCl (sodium chloride, 99.5%), KI (potassium iodide, 99%), as well as Na_2_S_2_O_3_ (sodium thiosulfate, 99%) were commercially available from Alfa Aesar, Thermo Fisher Scientific (China) Co. Ltd., Shanghai, China. Cement (P·O 42.5) was purchased from the China National Academy of Building Materials Science Co., Ltd. (Beijing, China), while the Chinese ISO standard sand produced according to GB/T 17671 to measure mortar strength was commercially obtained from Xiamen ISO Standard Sand Co. Ltd., Xiamen, China. Distilled water was prepared in our laboratory.

### 3.2. Instruments

The cement and additives were mixed using a cement paste mixer, NJ-160A. Both the initial setting time (IST) and final setting time (FST) of cement paste were tested on a Vicat apparatus. Cement mortar was prepared using a cement mortar mixer, JJ-5. The three instruments were all manufactured by Wuxi Xiyi Building Material Instrument Factory (Wuxi, China).

Both the compressive and flexural strengths of the cement mortar were tested using a fully automatic anti-folding and compression testing machine (WAY-300B) equipped with an automatic pressure testing machine control system (EHC-2300), with a maximum power of 300 kN and a pressing speed of 48 N s^−1^. The cement mortar was incubated in a numerical control standard cement conservation box (HBY-40B) at a temperature of 20 °C and a humidity of 90%. The two machines were manufactured by Wuxi Xiyi Building Material Instrument Factory.

FT-IR spectra were obtained using KBr pellets on a Bruker Tensor 27 spectrometer (Billerica, MA, USA). X-ray photoelectron spectroscopy (XPS) was carried out on a Kratos Axis Ultra DLD (Kratos Co., Ltd., Manchester, UK), using monochromatic Al-Kα X-rays (1486.6 eV) as a lighting source. The binding energy scale was calibrated by setting the C 1s peak to 284.8 eV as standard. The peaks were fitted using the Gaussian–Lorentz (G/L) product function with a 30% Lorentzian ratio. The wide-angle (2*θ* = 10–80°) X-ray diffractions of samples were obtained on a Philips X’Pert Pro diffractometer (PANalytical B.V. Co., Ltd., Almelo, The Netherlands) with Cu-Kα radiation (λ = 1.5418 Å), in association with an interval of 0.05° s^−1,^ was used as the X-ray source.

Inductively Coupled Plasma Optical Emission Spectrometer (ICP–OES) was performed on Agilent 5110 (Santa Clara, CA, USA), with pump rate of 60 r min^−1^, plasma gas of 12.0 L min^−1^, nebulizer flow of 0.70 L min^−1^, stable time of 20 s, auxiliary gas of 1.0 L min^−1^, reading access time of 5 s, sample flush time of 20 s, RF power of 1250 w. Thermogravimetric analysis (TGA) of the prepared sample was performed on a NETZSH TG 209C (Haimhausen, Germany), featuring a TASC 414/4 controller under N_2_ protection, where a heating rate of 10 °C min^−1^ was selected at 30–600 °C. Scanning electron microscopy (SEM) was carried out using Zeiss Sigma300 (Jena, Germany).

### 3.3. Synthesis of Block Copolymers

As shown in Figure 1a, AA (100 g, 1.38 mol), AMPS (100 g, 0.48 mol), and *n*-BuOH (200 mL, 162.96 g, 2.19 mol) were combined with distilled H_2_O (400 mL) into a three-necked bottle (1 L) having condenser, addition funnel and magnetic stirrer. Under vigorous stirring at 25 °C, (NH_4_)_2_S_2_O_8_ solution (2 g, 8.6 mmol, dissolved in 100 mL of distilled H_2_O) was added slowly through an addition funnel (within 1 h). Then, the temperature was slowly increased to 70 °C under continuous stirring, and the resulting mixture was stirred at 70 °C for 6 h. After cooling in air, the solvent was completely removed by rotary evaporation, and the remaining slightly yellow oil was obtained as AA–AMPS (**B1**).

As shown in Figure 1b, AA (100 g, 1.38 mol), AMPS (100 g, 0.48 mol), MAH (100 g, 1.01 mol), and *n*-BuOH (200 mL, 162.96 g, 2.19 mol) were combined with distilled H_2_O (400 mL) into a three-necked bottle (1 L) featuring condenser, addition funnel and magnetic stirrer. The subsequent process was the same as that for **B1**. The resulting AA, –AMPS–MA (**B2**), was also a slightly yellow oil.

### 3.4. Determination of Monomer Conversion of Block Copolymers

The bromine concentration (*X*, mg g^−1^, bromine consumption per gram of sample) was determined using the Chinese standard GB/T 10535-1997. In principle, Br_2_ (generated in situ) was covalently added to the unpolymerized monomers in the tested sample. The excessive Br_2_ would react with KI solution that was added subsequently, and the precipitated I_2_ was determined by the standard Na_2_S_2_O_3_ titration.

In practice, **B1** (or **B2**, 0.5000 g) was added to a volumetric flask (250 mL), and distilled H_2_O was added to a fixed volume. The resulting solution (25.00 mL) was transferred to an iodine flask (250 mL), and then a KBrO_3_–KBr mixed solution (10 mL, prepared by mixing 5.5 g of KBrO_3_ and 20.0 g of KBr with enough distilled H_2_O in a brown volumetric flask of 1000 mL was added. After shaking for 5 min, H_2_SO_4_ solution (20 mL, 3 mol L^−1^) was introduced, and HgSO_4_ solution (5 mL, prepared by mixing 15 g of HgSO_4_ with 14 mL of concentrated H_2_SO_4_ into 475 mL of distilled H_2_O) was further added. The resulting solution was shaken well and then stored in a dark place for 30 min at 0–20 °C.

Next, NaCl solution (15 mL, 116 g L^−1^) and KI solution (10 mL, 100 g L^−1^) were added, and the resulting solution was shaken well and stored in a bright place for 5 min at room temperature. Then, distilled H_2_O (20 mL) was added. The solution obtained so far was titrated with standard Na_2_S_2_O_3_ solution (0.1 mol L^−1^) until the solution color became slightly yellow. Then, a starch indicator solution (1 mL, 10 g/L) was added, and titration was continued until the color of the solution changed from blue to colorless. The bromine number (*w*, mg g^−1^) was calculated according to Equation (1):

(1) $$X=\frac{c\times (V_{0}-V)\times 0.0799\times 1000}{m}$$

where *X is* the bromine number (mg g^−1^), *c* is the real concentration of the standard Na_2_S_2_O_3_ solution (mol L^−1^); *V*_0_, consumed volume of the standard Na_2_S_2_O_3_ solution in the sample blank experiment, *V*, is the consumed volume of the standard Na_2_S_2_O_3_ solution in the regular experiment; 0.0799, is the bromine (Br) mass (g) derived from the consumption of the ideal Na_2_S_2_O_3_ solution (1 mL, 1.000 mol L^−1^), and *m*, is the mass of the tested sample.

The monomer conversion (*α*, %) of the tested sample was calculated according to Equation (2):

(2) $$\alpha =1-\frac{Xm_{0}}{1000\times M\times (m_{1}/M_{1}+m_{2}/M_{2}+m_{3}/M_{3})}$$

where *α* is the monomer conversion of the tested sample; *X* is the bromine number (mg g^−1^); *m*_0_, *m*_1_, *m*_2_, *m*_3_, corresponding to the masses of all monomers, AA, AMPS, MA (blank for **B1**), respectively; and *M*, *M*_1_, *M*_2_, and *M*_3_ are the relative molecular weights of Br, AA, AMPS, and MA (blank for **B1**).

### 3.5. Synthesis of Setting Accelerators

At room temperature (20–30 °C), **B1** (or **B2**, 100 g) and AS (350 g) were mixed with distilled water (150 g) in a three-necked bottle (1 L) with mechanical stirring equipment. After vigorous stirring at room temperature for 1 h, the resulting white emulsion was decanted and stored for future use, corresponding to the accelerators **B1a** and **B2a**.

### 3.6. Measurement of Setting Time

On the basis of the Chinese standard JC 477-2005, both the IST and FST of cement paste were measured on a Vicat apparatus as follows. At first, cement (400 g) was combined with distilled H_2_O (148 g for an accelerator dosage of 6 wt.% over cement, 144 g for that of 7 wt.%, 140 g for that of 8 wt.%) into a cement paste mixer, which was first stirred at low speed for 30 s. Second, the accelerator (**B1** or **B2**; 24 g, dosage of 6 wt.% over cement; 28 g, 7 wt.%; 32 g, 8 wt.%) was introduced, which was initially stirred at low speed for 5 s, then at high speed for 15 s. Furthermore, the total cement paste was quickly poured into a round mold, crushed, and then slightly vibrated. The paste surface was further smoothed by using a scraper.

Both the IST and FST were measured every 10 s over the Vicat apparatus by penetrating a needle of a fixed cross-section into the cement paste under constant force. The IST was derived from the time between releasing the needle in a free fall manner and the needle reaching the preset depth (4 ± 1 mm from the bottom). The FST was calculated as the time between the endpoint of the IST and the moment at which the needle could no longer drop.

### 3.7. Measurement of Compressive and Flexural Strengths

The compressive and flexural strengths of the cement mortar were also tested according to the Chinese standard JC 477-2005. First of all, cement (900 g) and distilled H_2_O (468 g at an accelerator dosage of 6 wt.% over cement, 459 g for 7 wt.%, 450 g for 8 wt.%) were combined into a mixing bowl, which was immediately stirred at low speed for 30 s over the assorted cement mortar mixer (JJ-5). After further stirring at a low speed for 30 s, Chinese ISO standard sand (1350 g) was added gradually. Next, the resulting mixture was mechanically stirred at a high speed for 30 s, paused for 90 s, and stirred again at a high speed for 30 s. Instantly after stirring, the accelerator (**B1** or **B2**; 54 g, dosage of 6 wt.% over cement; 63 g, 7 wt.%; 72 g, 8 wt.%) was introduced, and the resulting mixture was further stirred at low speed for 5 s, then at high speed for 15 s. Furthermore, the cement mortar was immediately transferred into a mold with the size of 40 mm × 40 mm × 160 mm (trial mold for cement mortar soft scouring), and then stored in a cement conservation box at 20 °C with a humidity of 90% for a pre-set incubation time (6 h, 24 h, 28 d, and 90 d).

## 4. Conclusions

In conclusion, the use of aluminum sulfate coordinated by binary and ternary water-soluble block copolymer appeared to be an efficient accelerator with a hardening effect for cement setting. The main conclusions are summarized as follows:

(1) The two block copolymers were prepared with high efficiency through ammonium persulfate-catalyzed free radical polymerization in an aqueous solution. The ternary block copolymer having monomers such as AA (acrylic acid), AMPS (2-acrylamido-2-methylpropane sulfonic acid), and MAH (maleic anhydride) showed higher monomer conversion than the binary copolymer featuring AA and AMPS units.
(2) The combination of aluminum sulfate with two synthesized block copolymers can significantly shorten the initial and final setting times of the cement paste and improve the compressive and flexural strengths of the mortar. The conjunction of aluminum sulfate with a ternary block copolymer (AA–AMPS–MAH) performed even better than that with a binary copolymer (AA–AMPS) in cementing.
(3) The role of synthesized block copolymers lay in the inhibition of Al^3+^ hydrolysis into unreactive Al(OH)_3_ or Al_2_O_3_. In particular, Al(SO_4_)(OH), derived from the mixing of Al^3+^ with AA–AMPS–MAH, was detected as an unexpected and highly active intermediate for optimizing cement hydration.
(4) The use of aluminum sulfate associated with AA–AMPS–MAH would produce two new phases, including Ca_1.5_SiO_3.5_·xH_2_O and Ca_2_(Si_9_Al_3_)O_24_·8H_2_O in cement mortar after 24 h incubation compared to that with AA–AMPS, which actually acted as a great linker to enhance the mechanical strength of the mortar. Furthermore, the combination of aluminum sulfate with AA–AMPS–MAH produced a much denser mortar (28 d) with better mechanical strength than that with AA–AMPS.

This work will contribute to the advancement of cement accelerators, particularly in their combined application in the engineering of sprayed concrete (shotcrete) for tunnel and roadway construction.

## Figures and Tables

**Table 1:** The bromine number (*X*) and monomer conversion (*α*) of tested sample ^a^.

Sample	*m* (g) ^b^	*V* (mL) ^c^	*X* (mg g^−1^) ^d^	*α* (%) ^e^
blank	-	20.60	-	-
**B1**	0.50	14.70	94.28	96.90
**B2**	0.50	16.50	65.52	97.62

^a^ As in Section 3.4; ^b^ Mass of tested sample; ^c^ Consumed volume of Na_2_S_2_O_3_ solution in titration; ^d^ Bromine number, determined by Equation (1); ^e^ Monomer conversion, by Equation (2).

**Table 2:** Setting time of cement paste and mechanical strength of cement mortar under different accelerators ^a^.

Entry ^a^	Accelerator (Dosage) ^b^	Setting Time (Min, Cement Paste) ^c^		Compressive Strength (MPa, Cement Mortar) ^d^			Flexural Strength (MPa, Cement Mortar) ^d^		
		Initial (IST)	Final (FST)	6 h	24 h	28 d	6 h	24 h	28 d
1	blank	28.50 ± 0.81 ^e^	39.75 ± 0.95	0.6 ± 0.02	4.5 ± 0.31	22.4 ± 0.45	0.3 ± 0.02	2.7 ± 0.15	10.9 ± 0.10
2	AS (7%)	18.75 ± 0.95	36.74 ± 1.87	1.1 ± 0.16	6.0 ± 0.25	24.9 ± 0.50	0.8 ± 0.11	2.9 ± 0.02	11.7 ± 0.22
3	**B1** (7%)	26.77 ± 0.17	35.65 ± 0.27	0.9 ± 0.04	6.2 ± 0.30	23.9 ± 0.41	0.4 ± 0.03	2.9 ± 0.20	11.9 ± 0.17
4	**B1a** (6%)	4.75 ± 0.25	8.30 ± 0.30	1.3 ± 0.03	7.4 ± 0.20	25.9 ± 0.37	0.8 ± 0.01	3.6 ± 0.21	12.8 ± 0.19
5	**B1a** (7%)	1.03 ± 0.02	1.95 ± 0.04	1.3 ± 0.04	10.7 ± 0.23	27.9 ± 0.60	0.9 ± 0.15	4.0 ± 0.32	13.6 ± 0.28
6	**B1a** (8%)	1.35 ± 0.02	2.18 ± 0.05	1.6 ± 0.07	9.8 ± 0.30	27.0 ± 0.53	0.2 ± 0.07	3.8 ± 0.26	12.1 ± 0.31
7	**B2a** (6%)	2.01 ± 0.17	3.08 ± 0.09	1.7 ± 0.05	7.8 ± 0.26	26.2 ± 0.49	1.5 ± 0.07	4.3 ± 0.20	13.9 ± 0.09
8	**B2a** (7%)	1.01 ± 0.02	1.66 ± 0.02	1.8 ± 0.03	12.5 ± 0.55	30.5 ± 0.67	1.7 ± 0.03	4.6 ± 0.11	14.5 ± 0.12
9	**B2a** (8%)	1.45 ± 0.05	2.02 ± 0.06	1.5 ± 0.03	9.8 ± 0.19	27.3 ± 0.39	1.1 ± 0.06	4.1 ± 0.08	12.9 ± 0.28

^a^ Experimental details as in Section 3.6 and Section 3.7; ^b^ Dosage of accelerator over cement, mass percentage, as in Section 3.6 and Section 3.7; ^c^ As in Section 3.6; ^d^ As in Section 3.7; ^e^ Data: average value ± SD (standard deviation).

**Table 3:** Comprehensive strength retention ratio after 28 and 90 days.

Entry ^a^	*R*_28_ (%) ^b^	*R*_r, 90_ (%) ^c^
5	125	107
8	136	198

^a^ Corresponding to entries in Table 2; ^b^ Retention ratio after 28 days, *R*_28_ = *f*_t, 28_/*f*_r, 28_ × 100%, *f*_t, 28_ means comprehensive strength of 28 d for the tested mortar sample (MPa), *f*_r, 28_ means comprehensive strength of 28 d for the standard mortar sample (MPa, entry 1, Table 2), according to the Chinese standard GB/T 35159-2017; ^c^ Retention ratio after 90 days, *R*_r, 90_ = *f*_t, 90_/*f*_r, 28_ × 100%, *f*_t, 90_ means comprehensive strength of 90 d for the tested mortar sample (MPa), *f*_r, 28_ means comprehensive strength of 28 d for the standard mortar sample (MPa, entry 1, Table 2), according to the Chinese standard GB/T 35159-2017.

**Table 4:** The composition of cement ^a^.

Composition	CaO	SiO_2_	Al_2_O_3_	Fe_2_O_3_	SO_3_	MgO	K_2_O	Na_2_O	Ignition Loss ^b^
Content (wt.%)	60.66	18.26	5.95	4.01	4.75	1.76	1.35	0.41	2.85

^a^ Chemical composition of cement (raw material) was determined by ICP–OES; ^b^ Ignition loss was detected according to GB/T 34231-2017.

**Table 5:** Binding energy and atomic composition of the elements on the sample surface (depth: 0–10 nm).

Entry	C (1s)	O (1s)	S (2p)	Si (2p)	K (2p)	Ca (2p) or N (1s)	Al (2p)
Cement ^a^	284.80 (40.84) ^b^	530.80 (30.70)	167.80 (0.55)	101.80 (6.54)	292.80 (10.29)	346.80 (9.36)	73.80 (1.73)
**B1a**	284.80 (56.59)	531.80 (30.24)	168.80 (9.14)	- ^c^	-	401.80 (0.11) ^d^	74.80 (3.92)
**B2a**	284.80 (57.29)	531.80 (31.76)	168.80 (7.18)	-	-	401.80 (1.59) ^d^	74.80 (2.17)
5 (24 h) ^e^	284.80 (29.85)	530.80 (40.12)	167.80 (1.30)	101.80 (9.55)	292.80 (7.52)	346.80 (11.51)	73.80 (0.15)
8 (24 h) ^e^	284.80 (29.27)	531.80 (39.89)	169.80 (0.89)	102.80 (9.22)	293.0 (7.37)	346.80 (11.35)	73.80 (2.01)

^a^ Raw material, as in Section 2.1; ^b^ Binding energy (eV), along with atomic percentage (at%) in parentheses; ^c^ Not found or not counted by instrument due to low content; ^d^ Binding energy (atomic percentage, at%) of N 1s photoelectron; ^e^ Cement mortar, corresponding to entries in Table 2.

## Data Availability

The raw data supporting the conclusions of this article will be made available by the authors upon request.
